# Preclinical Evaluation of a Novel 3D-Printed Movable Lumbar Vertebral Complex for Replacement: In Vivo and Biomechanical Evaluation of Goat Model

**DOI:** 10.1155/2021/2343404

**Published:** 2021-12-10

**Authors:** Feng Zhang, Jiantao Liu, Xijing He, Rui Wang, Teng Lu, Ting Zhang, Zhiyu Liu

**Affiliations:** ^1^Department of Orthopedics, Second Affiliated Hospital of Xi'an Jiaotong University, Xi'an, Shaanxi Province, 710004, China; ^2^Department of Orthopaedics, Xi'an Fourth Hospital, Xi'an, Shaanxi Province, 710004, China; ^3^Department of Orthopaedics, First Affiliated Hospital of Xi'an Jiaotong University, Xi'an, Shaanxi Province, 710061, China; ^4^Department of Orthopedics, Xi'an International Medical Center Hospital, Xi'an Shaanxi Province, 710100, China

## Abstract

**Purpose:**

This was an in vivo study to develop a novel movable lumbar artificial vertebral complex (MLVC) in a goat model. The purpose of this study was to evaluate clinical and biomechanical characteristics of MLVC and to provide preclinical data for a clinical trial in the future.

**Methods:**

According to the preoperative X-ray and CT scan data of the lumbar vertebrae, 3D printing of a MLVC was designed and implanted in goats. The animals were randomly divided into three groups: intact, fusion, and nonfusion. In the intact group, only the lumbar vertebrae and intervertebral discs were exposed during surgery. Both the fusion and nonfusion groups underwent resection of the lumbar vertebral body and the adjacent intervertebral disc. Titanium cages and lateral plates were implanted in the fusion group. MLVC was implanted in the nonfusion group. All groups were evaluated by CT scan and micro-CT to observe the spinal fusion and tested using the mechanical tester at 6 months after operation.

**Results:**

The imaging results showed that with the centrum, the artificial endplates of the titanium cage and MLVC formed compact bone trabeculae. In the in vitro biomechanical test, the average ROM of L3-4 and L4-5 for the nonfusion group was found to be similar to that of the intact group and significantly higher in comparison to that of the fusion group (*P* < 0.05). The average ROM of flexion, extension, lateral bending, and rotation in the L2-3 intervertebral space significantly increased in the fusion group compared with the intact group and the nonfusion group (*P* < 0.001). There were no significant differences in flexion, extension, lateral bending, and rotation between the nonfusion and intact groups (*P* > 0.05). The average ROM of flexion, extension, lateral bending, and rotation in the L2-5 intervertebral space was not significantly different between the intact group, the fusion group, and the nonfusion group, and there was no statistical significance (*P* > 0.05). HE staining results did not find any metal and polyethylene debris caused by abrasion.

**Conclusion:**

In vivo MLVC can not only reconstruct the height and stability of the centrum of the operative segment but also retain the movement of the corresponding segment.

## 1. Introduction

Traditional anterior lateral vertebrectomy and fusion is used to treat spinal tuberculosis, severe fracture, and tumors. It plays a major role in achieving spinal cord decompression, reconstruction of vertebral height, and spinal stability of the lumbar spine [1–5]. However, it also has some disadvantages, such as the loss of motion of the lumbar spine in the surgical area and the postoperative biomechanical changes of the lumbar spine lead to accelerated degeneration of adjacent segments [6–8]. Nonfusion technology maintains or restores intersegmental motion, reduces adjacent segment degeneration, and achieves the effect of protecting spinal motion [9]. Although nonfusion prostheses such as artificial intervertebral discs circumvent the concept of fusion, the vertebral height cannot be reconstructed. Nonetheless, the movable artificial lumbar vertebral complexes (MLVC) can overcome the above shortcomings. Currently, only a few studies have reported the research and development of MLVC [10, 11].

Thus, we have designed a MLVC that can not only reconstruct the height of the vertebral body but also imitate the intact motion of the lumbar spine. In order to evaluate the biocompatibility and mechanical properties of the prosthesis, a biomimetic lumbar complex was manufactured by 3D printing, and animal experiments were conducted to lay a foundation for future clinical experiments. In this study, we used goats bearing similar physiological functions to human lumbar to construct the movable lumbar model. If the MLVC study is successful, its clinical application will enable patients with lumbar disease to retain intervertebral mobility and reduce the occurrence of degeneration in the adjacent intervertebral spaces.

## 2. Materials and Methods

### 2.1. Specimen Screening and Pretreatment

This study was approved by the Ethics Committee of the Xi'an Jiao Tong University Health Science Center (ethics no. 2017025). 18 Boer goats aged 1-2 years, weighing 35–45 kg, were selected for the study (provided and maintained by the Animal Experimental Center, Xi'an Jiao Tong University Health Science Center). X-ray (NOVA FA-C Digital X-ray Photography System, SEDCAL, ES) and three-dimensional reconstruction of CT scan (64-slice, Discovery CT750, GE Healthcare, Milwaukee, WI, USA) for the lumbar spine were conducted before the operation, and those with spinal deformities, fractures, and destructive diseases were excluded. Finally, 18 goats were randomly and equally divided into three groups: intact, nonfusion, and fusion.

### 2.2. Anatomical Data Measurement

The resolution of CT scan image was set as 18.2 cm of display field, 256 × 165 of reconstruction matrix, and 256 × 171 of display matrix. CT scanned images were saved in DICOM format, and a total of 1359 DICOM images were obtained. The images were imported into the Mimics software (Version16.0, Materialise NV, Leuven, Belgium), and the CT images were thresholded and segmented according to the gray value of the images. The Calculate 3D module in the software was used to perform three-dimensional reconstruction and generate a three-dimensional model of the lumbar spine. The L4 vertebral body and adjacent intervertebral disc were measured by the measurement tool on the software. The measurement indexes included anterior height, middle height, and posterior height of vertebral body; the median sagittal diameter and median coronal diameter of the superior, middle, and inferior planes of the vertebral body; and the anterior height, middle height, and posterior height of the L3-L4 and L4-L5 intervertebral discs.

### 2.3. Design and Processing of Prothesis

**Figure 1:**
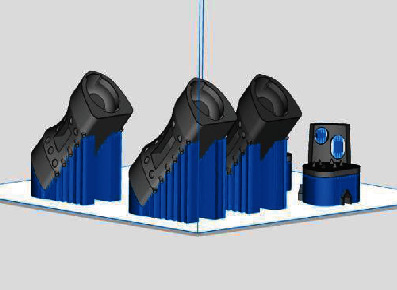
Modeling of prosthesis (Processed by SolidWorks).

**Figure 2:**
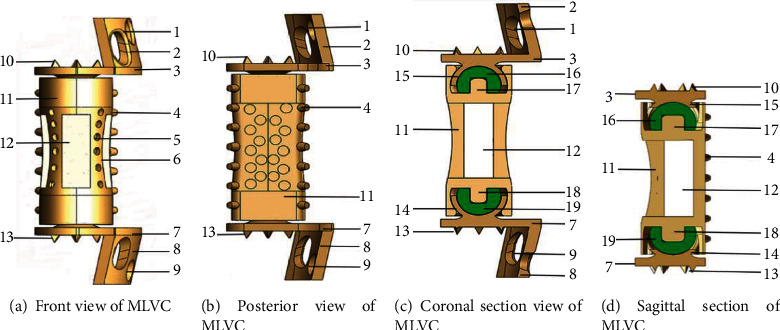
Schematic and section of MLVC (Processed by SolidWorks). (1) Upper screw hole. (2) Upper lateral plate. (3) Upper artificial endplate. (4) Protruding pillars. (5) Cone-like depressions. (6) Window of bone graft. (7) Lower artificial endplate. (8) Lower lateral plate. (9) Lower screw hole. (10) Upper conical thorn. (11) Vertebral body component. (12) Bone graft warehouse. (13) Lower conical thorn. (14) Lower artificial metal mortar cup. (15) Upper artificial metal mortar cup. (16) Upper high cross-linked polyethylene hemisphere. (17) Upper central column. (18) Lower central column. (19) Lower high cross-linked polyethylene hemisphere.

According to the parameters of the goat lumbar spine and intervertebral disc, different goat lumbar parts were designed individually (Figure 1) by using SolidWorks 2017 software (Dassault Systèmes, USA). MLVC prosthesis is divided into the lumbar vertebral body components (Figure 2(11)) and upper and lower artificial endplate components (Figures 2(3) and 2(7)). The vertebral body is similar in shape to the goat lumbar vertebra and is composed of cylinder-like geometry. Due to the individual size difference of the goat lumbar spine, the size of prosthesis components is different. The cross-sectional area of the upper and lower planes of the vertebral body component ranges from 270.2mm^2^ to 183.52mm^2^, and the height of the vertebral body component ranges from 30 mm to 38 mm. Its interior is designed as a bone grafting bin, which is cuboid and hollow in structure (Figure 2(12)). There is a rectangular window (Figure 2(6)) on the left and right sides and in the middle of the bin. The three rectangular bone grafting windows are 15 mm in width and 41 mm in length, which is convenient for bone grafting from the windows. There are many small protruding pillars (Figure 2(4)) and cone-like depressions (Figure 2(5)) on the surface of the vertebral body assembly. There is a concave disc at each end of the vertebral body and the central column (Figures 2(17) and 2(18)) is a cylinder with a diameter of 1.5 mm and a height of 5 mm, and its function is to embed an arc-shaped lining to form a convex hemisphere.

The artificial endplate assembly consists of an artificial endplate, an arc-shaped side plate, and an artificial mortar cup (Figures 2(14) and 2(15)). All the six conical spines (Figures 2(10) and 2(13)) on the artificial endplate are 2 mm high, and the curved side plates are printed according to the lateral radian of the lumbar vertebrae. The height of the curved side plate is generally one-third of the height of the goat vertebral body, and the width is one-fourth of the circumference of the lateral edge of the vertebral body, so that it can completely match the anatomical shape of the goat's lumbar spine. The diameter of the two screw holes (Figures 2(1) and 2(9)) in the lateral plate (Figures 2(2) and 2(8)) is 4.2 mm, and the intervertebral disc assembly is fixed on the sides of the upper and lower vertebral bodies with two screws with a diameter of 4.0 mm. In order to produce mobility at the level of intervertebral space, we designed the artificial joint structure, in which the concave semispherical design with bilateral wing structure constitutes the artificial mortar cup. The artificial mortar cup is embedded with the convex hemisphere structure (Figures 2(16) and 2(19)) to form a ball and socket joint structure with mobility function. The concave hemisphere has a double flank structure with a length of 6 mm, a width of 4 mm, and a thickness of 1.5 mm, while the convex hemisphere has a diameter of 10 mm and a thickness of 1 mm. The concave hemisphere and the convex hemisphere are combined to form a movable sphere structure. This composite structure of intervertebral disc joint can not only exert the function of intervertebral disc movement but also enhance the stability of the whole complex.

**Figure 3:**
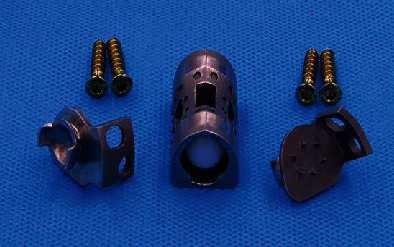
3D-printed component of MLVC.

In the software, the models of vertebral body parts and endplate parts were translated and rotated to couple them together. The lumbar spine STL model generated in Mimics software is imported into SolidWorks. In SolidWorks software, the MLVC prosthesis model was inserted between L3 and L5 vertebral bodies, and Boolean operation was performed to ensure that there was no gap or overlap between the prosthesis model and lumbar model so as to ensure the contact relationship of each structure. Then, MLVC was optimized by Materialise Magics software (Version 21.0, Materialise NV, Leuven, Belgium). The Materialise Build Processor software (Version1.2, Materialise NV, Leuven, Belgium) was used to transform these designs into working files. Subsequently, selective laser melting (SLM) technology was applied for 3D printing of a goat lateral plate and metal components of a MLVC (Figure 1). These metal parts were printed in 3D by Xi'an Bright Laser Technologies Co., Ltd. (BLT) using Ti6A14V powder (3D printer is BLT-S200 produced by Xi'an BLT Laser Technologies Co., Ltd.). The hemispheric structure was manufactured by Beijing Chunlizhengda Medical Instruments Co., Ltd. using high cross-linking polyethylene. In addition, the postprocessing, polishing, and assembling of spherical fossa joints were conducted by Beijing Fule Science & Technology Development Co., Ltd. (Figures 2 and 3).

### 2.4. Anesthesia Operation and Postoperative Management

#### 2.4.1. Anesthesia Mode

Goats were intravenously injected with propofol at a dose of 4 mg/kg for anesthesia. During the operation, the anesthesia was maintained by intravenous injection of pentobarbital (30 mg/kg) [6].

#### 2.4.2. Surgical Procedures

Goats were randomly and equally divided into the intact, fusion, and nonfusion groups. At 30 min before the operation, the goats were treated with an atropine sulfate injection (0.02 mg/kg) [12] and an intravenous drip of cefazolin sodium (25 mg/kg). Spinal nerve monitoring equipment (Protektor32 IOM, XLTEK, USA) was installed to record somatosensory evoked potentials (SEP), motor evoked potentials (MEP), and electromyogram (EMG) of the lower extremities. Different stimulation intensities, wave widths, and stimulation frequencies were set for SEP (15-20 mA, 10 ms, and 1-5 Hz) and MEP (30-35 mA, 50 ms, and 300-400 Hz), respectively.

The operative region was disinfected by Entoiodine. A left lumbar retroperitoneal approach was applied at about 3 cm from the midline of the spine. A longitudinal incision, approximately 15 cm in length, was made at the center of the L4 vertebral body. Skin and subcutaneous tissues were cut open, and the lumbar spine was exposed. After douching, the goats in the intact group were sutured layer by layer, sterilized, and bandaged.

**Figure 4:**
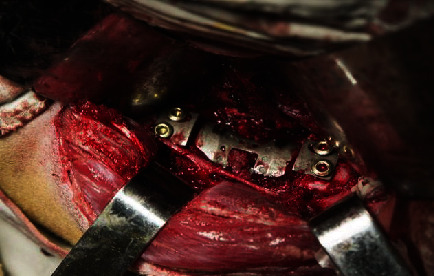
A MLVC was implanted into the lumbar spine of a goat.

In the nonfusion group, the upper and lower intervertebral discs of the L4 vertebral body were resected. The L4 vertebral body was excised by a grinding drill, bone chisel, and bone-biting forceps, and the ventral to anterior longitudinal ligaments were preserved. The right cortex and the posterior cortex of the L4 vertebral body were preserved. The lower endplate of L3 and the upper endplate of L5 were partly stricken off. The bone debris was inserted into the artificial vertebral body; the prosthesis was compressed, filled, assembled, and implanted into the vertebral defect area. The artificial endplate was attached to the upper and lower vertebral bodies and then fixed with four screws (Figure 4). The wound area was sutured as the intact group.

**Figure 5:**
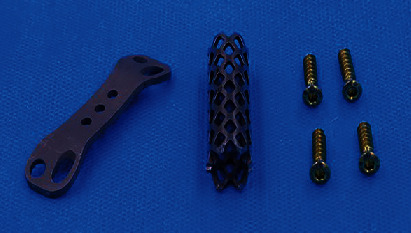
Titanium cage and 3D-printed plate.

**Figure 6:**
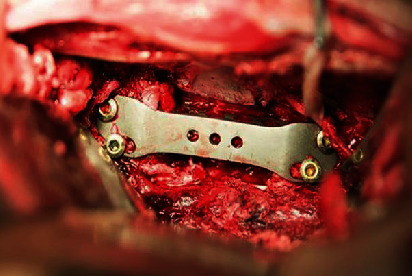
A titanium cage and 3D-printed plate were implanted into lumbar spine of goat.

In the fusion group, the resection of the vertebral body and intervertebral disc was conducted similar to that of the nonfusion group. The bone debris was placed into an appropriate titanium cage, pressed, and filled. The 3D-printed titanium alloy plate (Figure 5) was placed on the lateral side of the lumbar spine. The plate and lumbar spine fit satisfactorily and were drilled sequentially, followed by insertion of the cancellous screw (Figure 6). The wounds were treated similar to that of the other groups.

X-ray and CT evaluation were performed after 6 months of the operation. Under anesthesia induced by pentobarbital sodium, potassium chloride solution was injected intravenously to execute the goats. L3–L5 segments and the surrounding muscle tissues were removed. The ligaments were preserved, and the biomechanical tests and histological observations were carried out.

### 2.5. Observation Indexes

#### 2.5.1. X-Ray and CT Measurements

Postoperative X-ray films of the lumbar spine in goats were used to evaluate the position of the prosthesis and titanium cage. Six months after the operation, the goat lumbar vertebrae were scanned by multislice spiral CT to observe the position of the implants. Bone graft fusion and fusion of the artificial endplate and vertebral body were observed by micro-CT. Magin and Delling's [13] method was used to evaluate the intervertebral fusion as follows: grade 0, no callus formation; grade 1, a little amount of callus formation; grade 2, moderate callus formation; grade 3, more callus formation; and grade 4, a large amount of callus formation.

### 2.6. Biomechanical Tests

**Figure 7:**
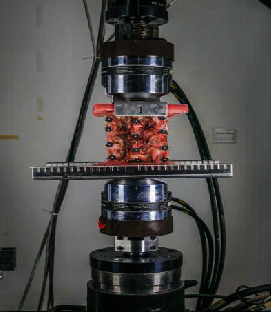
Mechanical testing of MLVC.

In vitro mechanical testing was performed using an MTS 858 Mini Bionix II Mechanical Tester (MTS, USA) in the National Key Laboratory of Mechanics, Xi'an Jiao Tong University. Because of the limitation of the length of the loading material, the goat lumbar vertebrae were only cut into L2–L5 segments. Three reflective marker points (3 mm in diameter) were fixed tightly to L2, L3, L4, and L5 each, with a total of 12 reflective points. The specimens were placed on a parallel bar hydraulic servocontrolled biomaterial tester, and the coding points and measuring scales were placed and fixed around the tester (Figure 7). The motion of the marker points of the vertebral body was recorded accurately by the photoelectronic three-dimensional motion capture system (MPS, ChenWei Inc., Zhengzhou, China).

To minimize the effect of tissue creep, each specimen was loaded and released three times, and only the latest data were used for statistical analysis. Each specimen was assessed three times, and the average was applied to reduce the experimental errors.

In this study, a nondestructive elasticity testing method was employed to measure biomechanical parameters simulating the physiological activities of the lumbar spine, where the lumbar spine specimens are allowed to produce flexion-extension and left-right rotation. A torque of 0, 1, 2, 3, and 4 Nm was gradually applied at the head of the specimen to realize flexion and extension, lateral bending, and rotation.

MTS 858 directly rotates the specimen and records the rotation angle. Therefore, the specimen was placed directly in the center of the load, and the maximum load was set at 4 Nm during the mechanical testing process. The machine increased the load at a speed of 0.01 Nm/s [14]. The spatial instantaneous position of the marker was recorded by the above device and converted into the motion range of the vertebral body by MPS software.

### 2.7. Safety of the Prosthesis Material

The goats were executed at 6 months after the operation. In all the three groups, muscle and fascial tissue, spleen, and adjacent lymph nodes of goats were removed. The extracted tissue was stained with HE to observe the metal and polyethylene debris at 200x under a microscope.

H&E staining was performed (Abcam, Cat. no. ab245880) according to the manufacturer's instructions. Briefly, the sections were deparaffinized and hydrated in distilled water, followed by incubation with hematoxylin for 5 mins. Subsequently, the sections were covered and incubated with adequate Bluing Reagent for 10–15 s, rinsed in distilled water then stained with eosin Y.

### 2.8. Statistical Analysis

All statistical analyses were carried out using SPSS 20.0 (IBM Co., USA) which was used for statistical analysis. The range of movement (ROM) is expressed as the mean value ± standard deviation. The range of movement (ROM) of each intervertebral space was analyzed by one-way ANOVA and multiple LSD comparisons. *P* < 0.05 indicates statistical significance.

## 3. Results

### 3.1. General Observation

**Figure 8:**
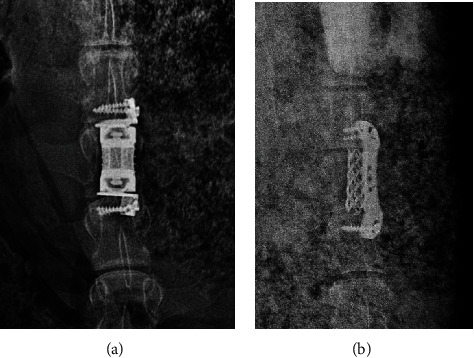
X-ray films of the goat lumbar spine after the operation in the nonfusion (a) and fusion (b) groups.

This study consisted of 18 goats, and the operation time was 1.5–3 (average, 2.26) h. The goats ate normally after awakening. The intraoperative bleeding volume was 450–740 (average, 576.5) mL in the goats. X-ray films of the lumbar spine showed that the prosthesis, the position of the titanium cage, and the plate were well fixed (Figure 8).

One goat died during the operation due to an anesthesia accident. The muscle strength of the left hind limb decreased in a goat after the operation, and 10 days later, it returned to normal.

**Figure 9:**
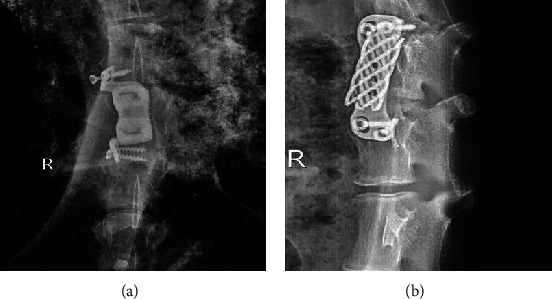
The implants were displaced after operation. (a) MLVC displacement and screw loosening occurred in the nonfusion group. (b) Titanium cage displacement occurred in the fusion group.

**Figure 10:**
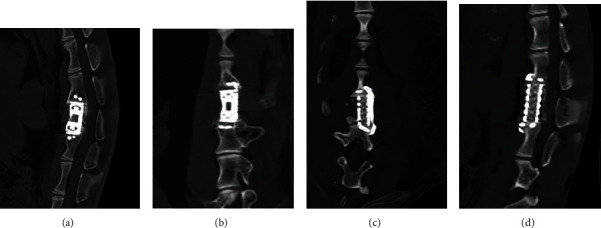
Sagittal (a.) and coronal (b.) CT images of the goat lumbar spine after the operation in the nonfusion group. Sagittal (c.) and coronal (d.) CT images of the goat lumbar spine at 6 months after the operation in the fusion group.

X-ray and CT scan for the lumbar spine were performed at 6 months after operation. In the nonfusion group, lumbar fusion occurred in 1 case. Moreover, in 1 case of the nonfusion group, the prosthesis was displaced, and the screws were loosened and withdrawn (Figure 9(a)). Titanium cage displacement occurred in 1 case of the fusion group (Figure 9(b)). CT scan of the lumbar spine showed that the implants were not displaced (Figure 10).

### 3.2. Callus Formation

**Figure 11:**
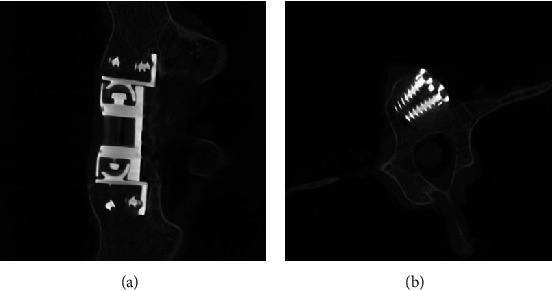
(a) The endplates of MLVC had obvious bone penetration (micro-CT). (b) There is significant bony ingrowth around the screw (micro-CT).

**Figure 12:**
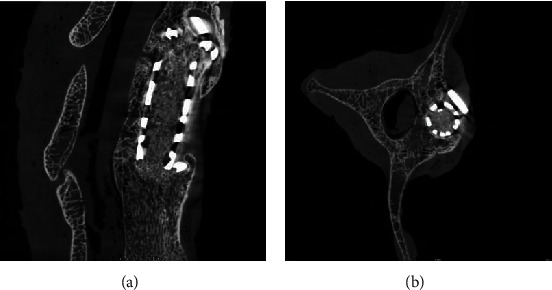
The titanium cage was fused with the vertebral bodies with callus formation (micro-CT).

Goats were executed at 6 months after the operation. Micro-CT of the lumbar spine showed callus formation in the fusion of the upper and lower endplates of the artificial prosthesis (Figure 11), titanium cage, and upper and lower vertebrae (Figure 12). In the fusion group, the titanium cage was fused with the upper and lower vertebral bodies with obvious callus formation. In the nonfusion group, the upper and lower endplates of MLVC had obvious bone penetration. There is also significant bony ingrowth around the screw.

### 3.3. Effect of MLVC on ROM

#### 3.3.1. ROM of L3-4 and L4-5

**Figure 13:**
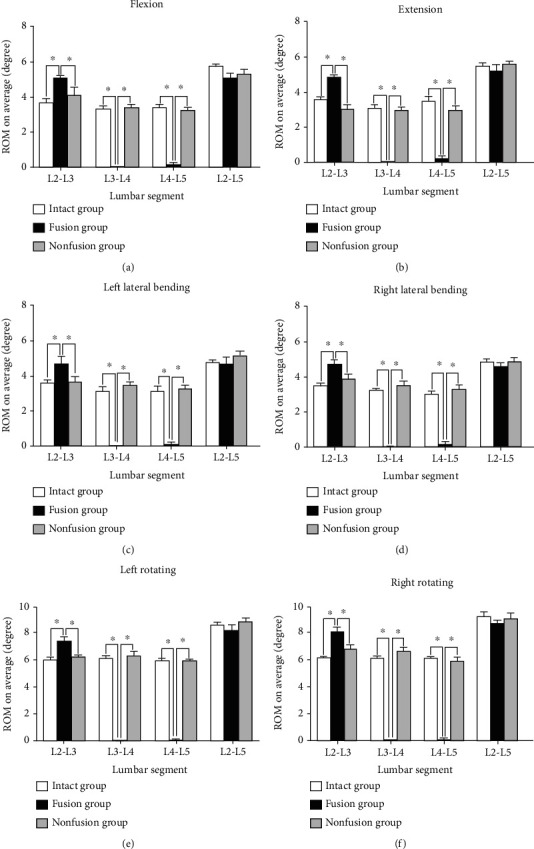
Statistical graph of the lumbar spine ROM of three groups. (a) ROM of flexion. (b) ROM of extension. (c) ROM of left lateral bending. (d) ROM of right lateral bending. (e) ROM of left rotating. (f) ROM of right rotating. ^∗^*P* < 0.05.

The results of the average ROM on L3-4 and L4-5 were similar in the intact groups and nonfusion groups, including the intervertebral space movement in flexion and extension, the left-right lateral bending, and left-right rotation. The average movement on these indexes of the fusion groups was significantly lower than that of the intact and nonfusion groups (*P* < 0.05) (Figure 13).

#### 3.3.2. ROM of L2-3

Compared to the other two groups (Figure 13), the average ROM of L2-3 was significantly higher in the fusion group in intervertebral space movement in flexion and extension, left-right lateral bending, and left-right rotation. In addition, no significant difference was detected in these indexes between the nonfusion and intact groups (*P* > 0.05).

#### 3.3.3. ROM of L2-5

As shown in Figure 13, no significant differences were detected on all six indexes among the intact, fusion, and nonfusion groups (*P* > 0.05).

In summary, all data in this detection presented that the characteristics of the nonfusion group were similar to that of the intact group, indicating that the nonfusion technology may preserve physiological function of the lumbar spine.

### 3.4. MLVC Is a Safe Prosthesis

**Figure 14:**
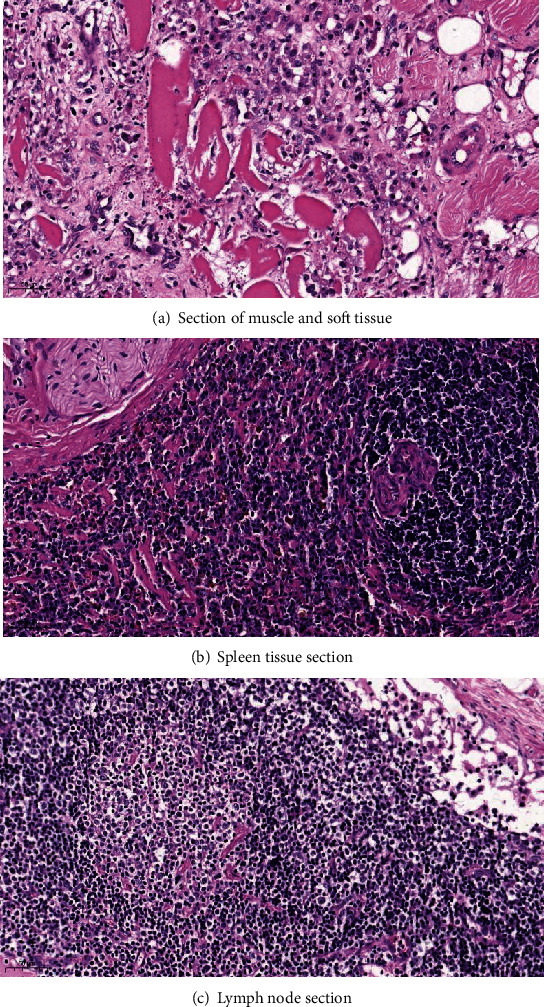
No foreign body debris was observed in the soft tissue, lymph nodes, and spleen surrounding MLVC (HE staining at 200x by microscopy).

Muscular and fascial tissues (Figure 14(a)), the spleen (Figure 14(b)), and adjacent lymph nodes (Figure 14(c)) around the prostheses in the goats were removed during the operation. No obvious inflammatory reaction was observed with HE staining at 200x by microscopy. As a result, no metal and polyethylene debris caused by prosthetic abrasion was found. These results indicated the safety of MLVC, which is suitable for clinical application.

## 4. Discussion

Before the application of a new bone substitute material, internal fixation material, and new technology in the orthopedic clinical field, animal models should be established to further study all kinds of indexes after spinal fusion. Only when the material is confirmed to be safe and not cause injury to animals, it can be utilized in a clinical study. The shape, size, and number of sheep and goat vertebral bodies are similar to those of the human vertebral body [15]. The sheep and goat model has been widely used in spinal experiments, especially in the study of cervical spondylosis and less in the research on lumbar vertebral disease. Reportedly, New Zealand white rabbits were used as animal spinal models with mortality and complications of >20% [16]. However, large animals as spinal models increase the complexity. As a large mammal, goat and sheep cannot walk upright like humans; nonetheless, some studies [15, 17] have compared their spines with those of humans. Although some morphological differences were detected between the lumbar spines, those of goat, sheep, and human were similar in physiological functions, and the lumbar spine of sheep and goat can be regarded as a spinal biomechanical model. In addition, according to the horizontal orientation of the vertebral trabeculae in goat and sheep, Smit [18] reported that the vertebral column of goats bears the same axial pressure as that of humans and can be used as an animal model for vertebral column implants. In the current study, because of the lack of experience in the first animal experiment and the significant weight of the goats, excessive use of narcotic drugs led to sudden cardiac arrest during the operation and death. One goat suffered from muscle weakness in the left hind limb after the operation, which was caused by nerve paralysis during the operation and reduced limb muscle strength after the operation, but myodynamia returned to normal gradually. One goat died of gastric reflux and aspiration after the operation. After dissection, some residual gastric pastes were observed in the bronchus. Therefore, we deduced that the obstruction of the bronchus by gastric contents after the operation was the cause of death. In the nonfusion group, lumbar fusion occurred in 1 case. In the fusion group, 1 case had titanium cage displacement. Because of excessive activity after the operation, we could not fix the external spine of the goats after the operation, which led to the displacement of the titanium cage and screw. Subsequently, the operations were completed in the other goats.

In this study, the spherical fossa-like joint of the intervertebral disc was designed by imitating the metal-high cross-linked polyethylene structure of the artificial joint. To avoid noise caused by metal collision and reduce the friction maximally, the contact surface was highly polished. The range of motion of the complex could fulfill the requirements of normal lumbar intervertebral disc motion in all directions theoretically. Conical thorns on the upper and lower endplates of the artificial vertebral body are inserted directly into the upper and lower vertebral bones, which in turn, increases the stability of the artificial vertebral body fixation that does not break or slip and leads to prosthesis displacement or instability. The integrity of the endplate is retained, the best interface between the inner plant, and the endplate is provided; meanwhile, the possibility of the inner plant falling into the adjacent vertebral body is reduced [19]. Since the artificial vertebral body is hollow and the pore design of the periprosthetic wall causes the debris embedded in the prosthesis to form bone fusion with the surrounding vertebral bone tissue and muscle-bone fusion with the surrounding soft tissue, long-term stability of the surgically treated diseased vertebra is ensured. Compared to the widely used artificial intervertebral disc, posterior dynamic stabilization system, and artificial vertebral body, the prosthesis can not only retain the motion function of the corresponding segments but also reconstruct the height and stability of the lumbar vertebrae. Previously, there were several types of artificial vertebral support designs, including metals, carbon fibers, and ceramics [20]. The prosthesis is composed of Ti6A14V and high cross-linked polyethylene, which are clinically common medical materials with sufficient histocompatibility [21]. The application of high cross-linked polyethylene is convenient. The use of cross-linked polyethylene mortar or lining in the joint prosthesis is not only familiar to the orthopedic surgeons but also does not require specific attention during the operation similar to that of learning new implant techniques. In addition, the application of metal-high cross-linked polyethylene has not given rise to concerns, such as the compatibility of the metal-metal prosthesis, such as the significant increase in the concentration of some metal ions in serum [22]. High cross-linked material has higher strength and resistance than ordinary polyethylene. Some studies have shown that the wear rate of the former is about 1/8 or 1/10 of that of the latter [23]. Muscle and fascia tissues, the spleen, and adjacent lymph nodes from around the goat prosthesis were removed during the operation. No visible inflammatory reaction was detected by HE staining, and no metal or polyethylene debris caused by prosthetic abrasion was found. The prosthesis not only has high-strength mechanical properties but also possess the advantages of nontoxicity, noncarcinogenesis, no distortion, and excellent histocompatibility. The characteristic of nonmagnetism does not influence the magnetic resonance examination and facilitates postoperative observation.

In theory, MLVC can move in the direction of flexion, extension, and lateral bending within ±5° [24]. In the intervertebral space movement of L3-4 and L4-5, the fusion group exhibited poor mobility than the nonfusion and intact groups, which could be caused by the fusion and fixation of the two segments. However, for intact and nonfusion groups in this study, it was found that the ROM of individual intervertebral space could hardly achieve 5° in some lumbar spine specimens of goats. This phenomenon could be because some soft tissues are not completely removed during soft tissue dissection. In the fusion group, we found that even if the L3-5 segment was fixed with a lateral plate, slight movement was noted in the L3-4 and L4-5 intervertebral spaces in some specimens. The three possible reasons were as follows: (1) Immediate movement of goats in the fusion group after the operation that was unable to achieve external fixation of the lumbar spine and restrict its movement; thus, the displacement of the titanium cage or plate appeared after the operation and led to poor osseointegration, resulting in fretting of the intervertebral space. (2) The lateral plate of the lumbar spine deformed slightly under torsion. (3) The fixation of four screws in the long segment is prone to produce a powerful force arm, which causes a certain range of motion in the fusion area. However, compared to the fusion group, the nonfusion group could significantly retain the L3-4 and L4-5 function of movement, and ROM was not significantly increased as compared to the intact group. After measuring ROM of the L2-3 adjacent segments, that in the fusion group was significantly higher than that in the nonfusion and intact groups; however, no significant difference was detected in ROM of L2-5 in the three groups. This result indicated that ROM of the original segment was redistributed to the adjacent segment after fusion, leading to increase in ROM of the adjacent intervertebral spaces. On the contrary, MLVC did not increase ROM of the adjacent segments while retaining the original segment's movement, which was considered while designing it. This ROM should not be wide, or else it might cause lumbar instability artificially. Prosthetic dislocation may occur in patients with severe lumbar instability after lumbar prosthesis replacement. To avoid such occurrences, the wing structure that we designed could prevent excessive slippage of the prosthetic spherical fossa joint and the artificial vertebral body. Since the vertebral body and intervertebral disc of goat spine are small, in order to avoid the influence of surgical scar on ROM of the lumbar spine, we set up the intact group. In different surgical groups, the influence of scar as a confounding factor on ROM of the lumbar spine can be avoided through the same surgical approach. The data of this study did not reveal a significant difference in the range of intervertebral motion between the nonfusion group and the same level in the segmentally intact group, suggesting that the lumbar spine of goat after implantation of MLVC had adequate stability, thereby laying a foundation for further clinical application.

Different from previous artificial vertebrae, the development of this prosthesis can not only reconstruct the height of intervertebral bodies but also imitate the movement function of the disc in the physiological position and minimize the degeneration of adjacent lumbar segments through nonfusion technology.

The current short-term study could not decipher whether there will be heterotopic ossification and spontaneous fusion around MLVC in the long term that leads MLVC to loss function of movement. However, 3D printing-customized prosthesis implantation is the direction of spinal surgery in the future. The problem of displacement, aseptic loosening, and fatigue resistance of MLVC will be resolved by 3D printing technology. Therefore, with the development of 3D printing technology, MLCV will play a crucial role in solving the problems of lossing motion from lumbar segment and adjacent segment degeneration after lumbar surgery.

## Data Availability

The data has been uploaded in the following supplemental files. The supplemental file does not support Excel, so other Excel data cannot be uploaded.

## Supplementary Materials

Supplementary Materials The average ROM on L3-4 and L4-5 was similar in the intact groups and nonfusion groups, including the intervertebral space movement in flexion and extension, left-right lateral bending, and left-right rotation. The average movement on these indexes of the fusion groups was significantly lower than that of the intact and nonfusion groups. The average ROM of L2-3 was significantly higher in the fusion group in intervertebral space movement in flexion and extension, left-right lateral bending, and left-right rotation. In addition, no significant difference was detected in these indexes between the nonfusion and intact groups
