# Supplementary material for: Preclinical Evaluation of a Novel 3D-Printed Movable Lumbar Vertebral Complex for Replacement: In Vivo and Biomechanical Evaluation of Goat Model
Source: Biomed Res Int. 2021 Dec 10;2021:2343404. doi: 10.1155/2021/2343404 (PMC8683181; doi:10.1155/2021/2343404)

L3-4ROM

| Category | Groups | | RD | SD | p value | 95% CI |  |
| --- | --- | --- | --- | --- | --- | --- | --- |
| Flexion ROM | **intact**  **intact** | **fusion** | 3.29 | 0.25 | 0.000 | 2.77, 3.82 |  |
|  |  | **non-fusion** | -0.08 | 0.25 | 0.760 | -0.60, 0.45 |  |
|  | **fusion** | **non-fusion** | -3.37 | 0.25 | 0.000 | -3.90, -2.84 |  |
| Extension ROM | **intact**  **intact** | **fusion** | 3.08 | 0.21 | 0.000 | 2.63, 3.53 |  |
|  |  | **non-fusion** | 0.16 | 0.21 | 0.457 | -0.29, 0.61 |  |
|  | **fusion** | **non-fusion** | -2.92 | 0.21 | 0.000 | -3.37, -2.47 |  |
| Left lateral bending ROM | **intact**  **intact** | **fusion** | 3.30 | 0.26 | 0.000 | 2.74, 3.86 |  |
|  |  | **non-fusion** | -3.32 | 0.26 | 0.241 | -0.88, 0.24 |  |
|  | **fusion** | **non-fusion** | -3.62 | 0.26 | 0.000 | -4.18, -3.06 |  |
| Right lateral bending ROM | **intact**  **intact** | **fusion** | 3.35 | 0.22 | 0.000 | 2.88, 3.83 |  |
|  |  | **non-fusion** | -0.29 | 0.22 | 0.213 | -0.77, 0.19 |  |
|  | **fusion** | **non-fusion** | -3.64 | 0.22 | 0.000 | -4.12, -3.17 |  |
| Left rotation ROM | **intact**  **intact** | **fusion** | 6.16 | 0.28 | 0.000 | 5.57, 6.75 |  |
|  |  | **non-fusion** | -0.17 | 0.28 | 0.549 | -0.76, 0.42 |  |
|  | **fusion** | **non-fusion** | -6.33 | 0.28 | 0.000 | -6.92, -5.74 |  |
| Right rotation ROM | **intact**  **intact** | **fusion** | 6.02 | 0.31 | 0.000 | 5.37, 6.68 |  |
|  |  | **non-fusion** | -0.46 | 0.31 | 0.153 | -1.11, 0.19 |  |
|  | **fusion** | **non-fusion** | -6.48 | 0.31 | 0.000 | -7.14, -5.83 |  |

L4-5 ROM

| Category | Groups | | RD | SD | p value | 95% CI |
| --- | --- | --- | --- | --- | --- | --- |
| Flexion ROM | **intact**  **intact** | **fusion** | 3.25 | 0.24 | 0.000 | 2.73,3.76 |
|  |  | **non-fusion** | 0.14 | 0.24 | 0.575 | -0.37,0.65 |
|  | **fusion** | **non-fusion** | -3.12 | 0.24 | 0.000 | -3.62,-2.60 |
| Extension ROM | **intact**  **intact** | **fusion** | 3.30 | 0.32 | 0.000 | 2.61,3.99 |
|  |  | **non-fusion** | 0.53 | 0.32 | 0.121 | -0.16,1.22 |
|  | **fusion** | **non-fusion** | -2.77 | 0.32 | 0.000 | -3.46,-2.08 |
| Left lateral bending ROM | **intact**  **intact** | **fusion** | 3.17 | 0.31 | 0.000 | 2.52,3.82 |
|  |  | **non-fusion** | -0.20 | 0.31 | 0.531 | -0.85,0.46 |
|  | **fusion** | **non-fusion** | -3.37 | 0.31 | 0.000 | -4.02,-2.71 |
| Right lateral bending ROM | **intact**  **intact** | **fusion** | 2.98 | 0.27 | 0.000 | 2.40,3.56 |
|  |  | **non-fusion** | -0.31 | 0.27 | 0.264 | -0.89,0.26 |
|  | **fusion** | **non-fusion** | -3.29 | 0.27 | 0.000 | -3.87,-2.72 |
| Left rotation ROM | **intact**  **intact** | **fusion** | 5.93 | 0.13 | 0.000 | 5.66,6.20 |
|  |  | **non-fusion** | 0.03 | 0.13 | 0.810 | -0.24,0.30 |
|  | **fusion** | **non-fusion** | -5.90 | 0.13 | 0.000 | -6.17,-5.63 |
| Right rotation ROM | **intact**  **intact** | **fusion** | 5.96 | 0.30 | 0.000 | 5.33,6.60 |
|  |  | **non-fusion** | 0.24 | 0.30 | 0.434 | -0.40,0.88 |
|  | **fusion** | **non-fusion** | -5.72 | 0.30 | 0.000 | -6.36,-5.09 |


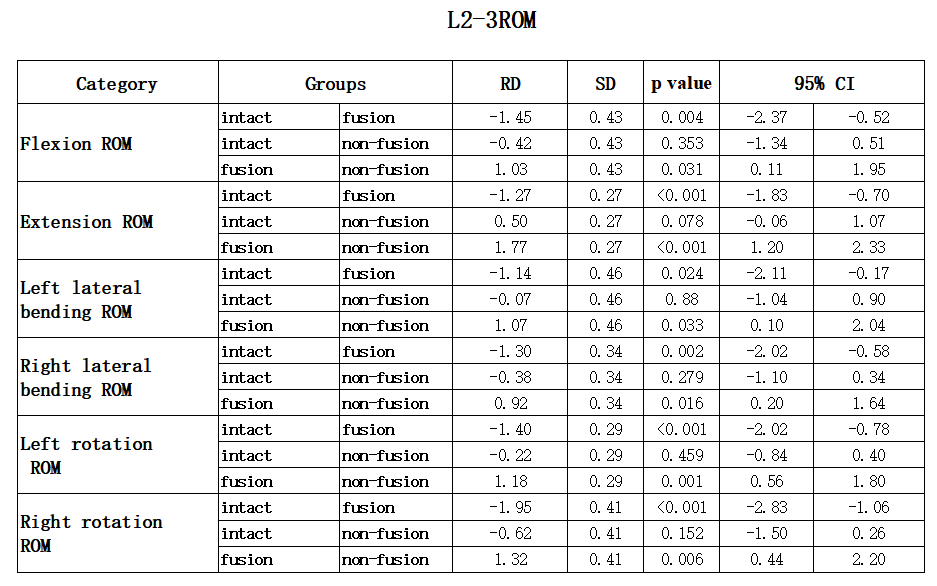

Supplement: Supplementary Materials — The average ROM on L3-4 and L4-5 was similar in the intact groups and nonfusion groups, including the intervertebral space movement in flexion and extension, left-right lateral bending, and left-right rotation. The average movement on these indexes of the fusion groups was significantly lower than that of the intact and nonfusion groups. The average ROM of L2-3 was significantly higher in the fusion group in intervertebral space movement in flexion and extension, left-right lateral bending, and left-right rotation. In addition, no significant difference was detected in these indexes between the nonfusion and intact groups [file 2343404.f1.docx]
